# Mapping implementation science in Pakistan’s health sector: a scoping review of frameworks, strategies, and implementation outcomes

**DOI:** 10.1080/16549716.2026.2732675

**Published:** 2026-09-16

**Authors:** Sheh Mureed, Saima Hamid, Sana Mangrio, Sumbal Hameed, Mehwish Javed

**Affiliations:** a Health Section, Ministry of Planning Development and Special Initiatives, Islamabad, Pakistan; b Program & Research Lead, Population Council, Islamabad, Pakistan; c Department of Community Medicine and Public Health Shifa College of Medicine, Shifa Tameer-e-Millat University, Islamabad, Pakistan; d Program Associate Research & Innovation Unit, National Emergency Operation Centre (NEOC), Islamabad, Pakistan; e Pediatrics Department, Women and Child Hospital, Adan Hospital, Al Ahmadi, Kuwait, Kuwait

**Keywords:** Implementation research, evidence synthesis, health systems, low- and middle-income countries, knowledge translation

## Abstract

This scoping review mapped how implementation science and implementation-relevant research have been used in Pakistan’s health sector. Searches of PubMed, Google Scholar, PakMediNet, WHO IRIS, and major Pakistani institutional repositories identified English-language studies published between 2010 and 2026 that examined implementation processes, strategies, determinants, or outcomes related to health interventions, programmes, or policies in Pakistan. Data were extracted using a structured template covering study characteristics, implementation frameworks, strategies, outcomes, contextual determinants, and actors. Descriptive and thematic synthesis was undertaken. A total of 111 studies were included. Publication activity increased markedly after 2020, and the evidence base was concentrated in maternal, newborn, and child health and infectious diseases. Facility- and district-level implementation settings predominated. Most studies referenced some type of framework, but relatively few used established implementation science frameworks such as the Consolidated Framework for Implementation Research or RE-AIM. Training, community engagement, and supervision were the most frequently reported implementation strategies. Feasibility, acceptability, appropriateness, and adoption were commonly assessed; sustainability was reported in about half of included sources, whereas fidelity, penetration, and cost were less consistently reported. Overall, the review suggests that implementation-oriented research is increasingly visible in Pakistan but often remains adjacent to formal implementation science theory. Greater use of explicit frameworks, broader attention to emerging health priorities, and stronger assessment of sustainability, scale, and costs could strengthen evidence-informed implementation and health system improvement in Pakistan.

## Paper Context

**Main findings**: Implementation-oriented research in Pakistan is increasing, but explicit application of established implementation science frameworks remains limited.

**Added knowledge**: This review maps frameworks, strategies, implementation outcomes, and actors across 111 studies and shows where implementation science is present in applied but often under-labelled forms.

**Global health impact for policy and action**: Stronger use of implementation science theory and better measurement of sustainability, scale, and costs could improve evidence-informed implementation and health system strengthening in Pakistan and similar lower-middle-income settings.

## Background

Implementation science has emerged as an important field for understanding how evidence-based health interventions can be translated into routine health system practice. Although advances in health sector research have produced a large body of effective interventions, a persistent gap remains between what is known to work and what is implemented in real-world settings. This ‘know–do gap’ represents a major challenge for health systems globally, particularly in low- and middle-income countries (LMICs), where institutional capacity, financing constraints, and sociocultural factors influence how interventions are delivered and sustained. Implementation science addresses this challenge by studying methods that promote the systematic uptake of research findings and evidence-based practices into routine care in order to improve the quality and effectiveness of health services [1,2].

Over the past two decades, the field has developed a range of conceptual frameworks and methodological approaches to analyze implementation processes. Frameworks such as the Consolidated Framework for Implementation Research (CFIR), Reach, Effectiveness, Adoption, Implementation, and Maintenance (RE-AIM), and the Active Implementation Frameworks (AIF) provide structured approaches for examining implementation determinants, strategies, and outcomes across health system contexts [3–5]. These frameworks emphasize that successful implementation depends not only on intervention effectiveness but also on organizational capacity, leadership, stakeholder engagement, and supportive policy environments. Implementation science therefore focuses on understanding how interventions interact with the complex systems of actors, institutions, and resources that shape service delivery.

The importance of implementation research has increasingly been recognized in global health policy and practice. International organizations, including the World Health Organization, have emphasized implementation research as a critical tool for strengthening health systems and accelerating progress toward universal health coverage and the Sustainable Development Goals [2]. Implementation science approaches are now widely applied to address challenges related to maternal and child health, infectious disease control, mental health integration, and non-communicable diseases. Concerns have also been raised that implementation science remains uneven in whose perspectives, leadership, and knowledge systems are represented globally, underscoring the importance of examining how implementation-oriented work is produced, labeled, and interpreted in LMIC contexts such as Pakistan [6].

Pakistan provides an important setting in which to examine this issue. With a population exceeding 240 million and a complex health system operating across national, provincial, and district levels, the country faces multiple public health challenges, including persistent maternal and child health concerns, infectious disease burdens, and a growing prevalence of non-communicable diseases. Nationally representative data highlight continuing health system challenges and inequalities in access to services across regions and populations [7]. Recent evidence from South Punjab illustrates the complexity of these challenges: socio-ecological factors shape mode of delivery and maternal outcomes [8,9], socioeconomic disparities constrain the implementation of evidence-based interventions such as Kangaroo Mother Care for preterm infants [10], and structural weaknesses in referral systems across levels of care limit effective implementation of health programmes [11]. At the same time, numerous national and provincial health initiatives have been implemented to address these challenges, often involving collaborations among government institutions, international donors, non-governmental organizations, academic partners, and community actors. However, it remains unclear how this body of work relates to the formal field of implementation science.

Despite this activity, the implementation science landscape in Pakistan has not been systematically mapped. It remains unclear which frameworks are guiding implementation work, whether established outcome domains are being measured, which actors and institutions are driving implementation efforts, and where the most critical evidence gaps lie. This absence of a comprehensive overview limits the ability of researchers, programme managers, and policymakers to identify where implementation science approaches could most strengthen health system performance in Pakistan and in comparable LMIC settings.

To better understand this landscape, this study conducts a scoping review of implementation science and implementation-relevant research in Pakistan’s health sector. The study aims to clarify how implementation science is currently represented in Pakistan’s health research landscape and to identify opportunities for strengthening the use of implementation science approaches in health system research and policy.

## Methods

This scoping review was conducted in accordance with the Preferred Reporting Items for Systematic Reviews and Meta-Analyses extension for Scoping Reviews (PRISMA-ScR). This scoping review used a structured approach to map how implementation science concepts and implementation-related research have been applied within Pakistan’s health sector. The PRISMA-ScR checklist is provided in Supplementary file S4. A protocol was developed a priori for this scoping review but was not formally registered.

A systematic search of the literature was conducted across multiple academic and institutional databases to identify relevant publications. The primary databases included PubMed, Google Scholar, PakMediNet, and WHO IRIS/EMRO, supplemented by searches of institutional repositories of major Pakistani health research institutions, including Aga Khan University (AKU), Health Services Academy (HSA), and the University of Health Sciences (UHS). Searches were conducted for studies published between January 2010 and June 2026, reflecting the period during which implementation science began to gain greater prominence in global health research.

Search strings combined implementation-related terms with health system and geographic identifiers. Key search terms included ‘implementation science,’ ‘implementation research,’ ‘implementation framework,’ ‘process evaluation,’ ‘implementation strategy,’ and ‘active implementation,’ combined with health-related terms such as ‘health,’ ‘healthcare delivery,’ or ‘health program,’ and the geographic identifier ‘Pakistan.’ Searches were adapted to the syntax requirements of each database. The full PubMed search strategy used for the review is provided in Supplementary File S3, and searches in other sources were adapted from the same core concepts using source-specific syntax. The complete database search strategies and supplementary extraction materials are provided in the supplementary files hosted on Zenodo (https://doi.org/10.5281/zenodo.22184997).

The strategy combined three concept blocks using the Boolean operator AND, with terms within each block combined using OR. Block 1 (implementation science concepts): (‘implementation science’[Title/Abstract] OR ‘implementation research’[Title/Abstract] OR ‘implementation framework’[Title/Abstract] OR ‘implementation strategy’[Title/Abstract] OR ‘implementation strategies’[Title/Abstract] OR ‘implementation outcome’[Title/Abstract] OR ‘implementation outcomes’[Title/Abstract] OR ‘implementation intervention’[Title/Abstract] OR ‘process evaluation’[Title/Abstract] OR ‘program implementation’[Title/Abstract] OR ‘programme implementation’[Title/Abstract] OR ‘knowledge translation’[Title/Abstract] OR ‘research utilization’[Title/Abstract] OR ‘research utilisation’[Title/Abstract] OR ‘evidence uptake’[Title/Abstract] OR ‘evidence-based practice’[Title/Abstract] OR ‘dissemination research’[Title/Abstract] OR ‘implementation fidelity’[Title/Abstract] OR ‘implementation sustainability’[Title/Abstract] OR ‘implementation feasibility’[Title/Abstract] OR ‘scale-up’[Title/Abstract] OR ‘scale up’[Title/Abstract] OR ‘quality improvement’[Title/Abstract] OR ‘active implementation’[Title/Abstract] OR CFIR[Title/Abstract] OR ‘Consolidated Framework for Implementation Research’[Title/Abstract] OR EPIS[Title/Abstract] OR ‘Exploration Preparation Implementation Sustainment’[Title/Abstract] OR PARIHS[Title/Abstract] OR ‘Promoting Action on Research Implementation in Health Services’[Title/Abstract] OR PRISM[Title/Abstract] OR ‘Practical Robust Implementation and Sustainability Model’[Title/Abstract] OR NPT[Title/Abstract] OR ‘Normalization Process Theory’[Title/Abstract] OR ‘RE-AIM’[Title/Abstract] OR ‘implementation science’[MeSH] OR ‘Diffusion of Innovation’[MeSH] OR ‘quality improvement’[MeSH] OR ‘Program Evaluation’[MeSH]). Block 2 (health system concepts): ‘health system’[Title/Abstract] OR ‘health sector’[Title/Abstract] OR healthcare[Title/Abstract] OR ‘health service’[Title/Abstract] OR ‘health programme’[Title/Abstract] OR ‘health intervention’[Title/Abstract] OR ‘primary care’[Title/Abstract] OR ‘public health’[Title/Abstract] OR ‘community health’[Title/Abstract] OR tuberculosis[Title/Abstract] OR HIV[Title/Abstract] OR ‘mental health’[Title/Abstract] OR immunization[Title/Abstract]. Block 3 (geography): Pakistan[Title/Abstract] OR Pakistan*[Title/Abstract] OR Pakistan[MeSH Terms]. Filters applied: English language; publication dates 1 January 2010 to 30 June 2026. Searches in Google Scholar, PakMediNet, WHO IRIS/EMRO, and institutional repositories were adapted from these same core concepts using source-specific syntax; Google Scholar was screened page by page to saturation (approximately 200 pages per search string).

Eligibility criteria were defined a priori using the Population, Concept, and Context (PCC) framework recommended by the Joanna Briggs Institute for scoping reviews [12]. Table 1 summarises the PCC-structured criteria applied during both screening stages.

**Table 1.** Eligibility criteria structured using the PCC framework.

PCC element	Inclusion criteria	Exclusion criteria
Population	Health sector actors and service recipients in Pakistan, including health workers, patients, community members, programme managers, and policymakers	Populations in non-health sectors (e.g. education, agriculture) or with no identifiable link to health service delivery
Concept	Studies addressing one or more of the following: use of an implementation science framework or model; implementation strategies (e.g. training, supervision, community engagement); implementation outcomes (e.g. acceptability, adoption, feasibility, fidelity, penetration, sustainability); barriers and facilitators to implementation; process evaluations; scale-up or sustainability of health interventions	Studies focused exclusively on clinical effectiveness, epidemiological surveillance, or disease burden without any implementation-related content; editorials, opinion pieces, and news articles
Context	Pakistan’s health sector at any level of the health system — community, primary, secondary, or tertiary care, district, provincial, or national level; Pakistan-only studies and multi-country studies providing Pakistan-specific data or analysis	Studies set exclusively in countries other than Pakistan; studies addressing non-health sectors; studies published before January 2010
Language and date	English-language publications; January 2010 to June 2026	Non-English publications; publications before January 2010

Two reviewers (SM and SH) independently screened the titles and abstracts against the eligibility criteria. Disagreements at this stage were resolved through discussion and consensus, with a third reviewer (SMur) consulted where agreement could not be reached. Full-text assessment of potentially eligible records was conducted by two reviewers (SM and SH), and data extraction was carried out by the same two reviewers using a standardised extraction template. A random sample of 20% of the extracted records was independently verified by a third reviewer (SMur) to ensure consistency. All reference management was performed using Mendeley (Elsevier, Amsterdam, Netherlands).

The extraction template captured bibliographic information, study characteristics, implementation science elements, and contextual factors influencing implementation. Variables extracted included author and publication year, study design, study setting, health domain, and study population. In addition, information was extracted on implementation frameworks or models used, implementation strategies described, and implementation outcomes reported. Implementation outcomes were categorized according to commonly used implementation science outcome domains such as acceptability, adoption, feasibility, fidelity, penetration, and sustainability [13]. The extraction framework also captured contextual determinants including reported barriers and facilitators.

The extracted data were analyzed using descriptive and thematic approaches. Descriptive analysis was used to summarize publication trends, study characteristics, and the distribution of implementation approaches across health domains. Thematic analysis was used to categorize implementation frameworks, strategies, and determinants of implementation described in the literature. Thematic analysis followed the framework analysis approach described by Ritchie and Spencer [14], applied through five analytical steps: (1) familiarisation, in which all extracted data were read in full to gain an overall understanding of the material; (2) identifying a thematic framework, in which a preliminary coding structure was developed by combining deductive categories drawn from the review objectives and established implementation science taxonomies with inductive themes emerging from the data; (3) indexing, in which each extracted record was systematically coded against the thematic framework; (4) charting, in which coded data were summarised in a matrix to allow comparison across studies and health domains; and (5) mapping and interpretation, in which patterns, relationships, and gaps were identified across the charted data to generate the thematic synthesis presented in the results.

In addition, the Active Implementation Framework (AIF) was applied post-hoc as an interpretive synthesis scaffold, rather than reflecting the framework used by the primary studies. This approach follows Fixsen and Fixsen [15], who demonstrated that AIF’s driver, stage, and systemic-change domains can successfully integrate diverse implementation frameworks – including CFIR – making it a suitable meta-framework for cross-tradition synthesis. This dual approach, framework analysis for systematic coding and AIF for interpretive synthesis, allowed both structured categorisation of extracted data and theoretically grounded interpretation of the Pakistan implementation science landscape.

Named implementation science frameworks included the Consolidated Framework for Implementation Research (CFIR), Reach, Effectiveness, Adoption, Implementation, and Maintenance (RE-AIM), Exploration, Preparation, Implementation, Sustainment (EPIS), Promoting Action on Research Implementation in Health Services (PARIHS), Practical, Robust Implementation and Sustainability Model (PRISM), Normalization Process Theory (NPT), and the Active Implementation Framework (AIF). Frameworks were coded as ‘other/local or adapted’ when studies reported a named or implicit organizing model, programme theory, conceptual approach, evaluation structure, or context-specific implementation framework that did not align with these predefined categories. For transparency, illustrative examples of studies classified as ‘other/local or adapted framework’ and the rationale for their classification are presented in Supplementary Table S2. Where studies were identified as protocols during data extraction, the methodology described within the protocol document (e.g. randomised controlled trial, mixed-methods study) was extracted and used as the basis for study design classification.

Generative AI assistance using ChatGPT (OpenAI, GPT-5) was used after author-led searching, screening, and data extraction to support the organisation and thematic categorisation of extracted data across the 111 included studies, and to assist with language editing and structural refinement of the manuscript. The tool was used because of the large volume of extracted data requiring systematic organisation across multiple coding categories. It was not used to generate original research data, search or screen studies, make eligibility decisions, interpret findings, or create figures or tables. All analytical decisions, coding verification, thematic interpretation, and final manuscript content were reviewed and approved by the authors. The following prompt was used to support the organisation of extracted data: ‘You are assisting with a scoping review of implementation science in Pakistan’s health sector. I will provide you with raw extracted data from individual studies. For each study, help organise the extracted text under the following categories: implementation framework used (if any); implementation strategies described; implementation outcomes reported; barriers to implementation; and facilitators of implementation. Do not add information not present in the extracted text. Flag any entries where categorisation is ambiguous.’ All coding decisions, thematic interpretation, and final synthesis were reviewed and verified by the authors.

Claude (Anthropic, Sonnet 5) was used, after all coding and analysis had been completed and verified by the authors, to conduct cross-tabulation analysis of implementation strategies and outcomes from the author-verified summary data. The tool was not used for any other part of the analysis, and all values displayed in Figure 5 were verified by the authors. The following prompt was used: ‘I have a table showing, for each of the six most frequently reported implementation strategies, the percentage of studies using that strategy that also reported each of the eight Proctor implementation outcomes. Please create a cross-tabulation figure (heatmap) visualising these percentages, with strategies and outcomes clearly labelled. Use only the values provided; do not add or infer any data.’

## Results

**Figure 1.**
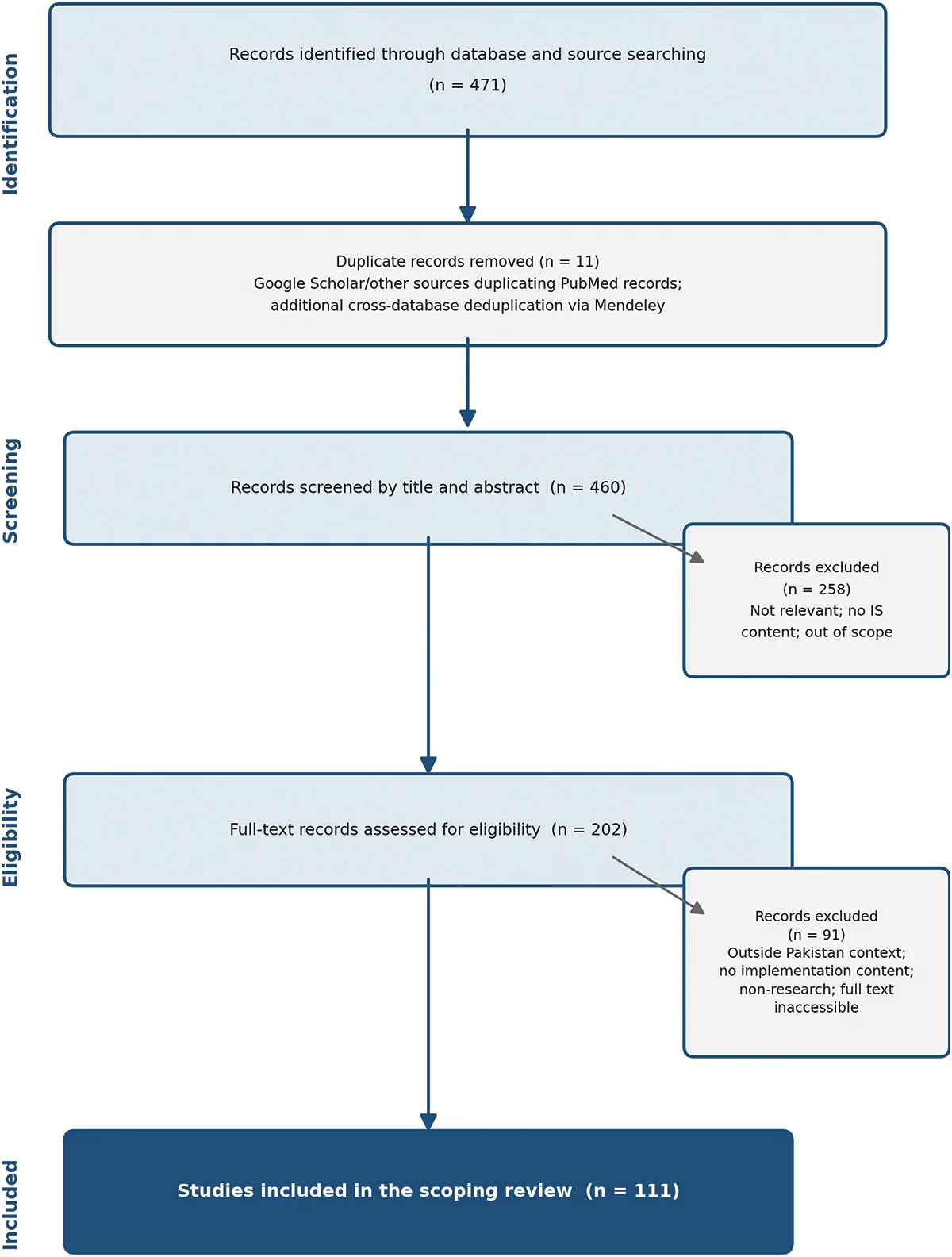
PRISMA-ScR flow diagram of study selection.

The literature search identified 471 records across all sources. Following removal of 11 duplicate records identified in the screening log and additional cross-database deduplication in Mendeley, 460 records were screened by title and abstract. Of these, 258 records were excluded for not meeting the eligibility criteria, leaving 202 full-text records to be assessed for eligibility. Following the restructured PCC eligibility criteria, 91 were excluded due to lack of relevance to Pakistan’s health sector, absence of implementation-related content, non-research publication type, or inaccessibility of full text. A total of 111 studies were included in the final scoping review (Supplementary Table S1). The study selection process is summarised in the PRISMA-ScR flow diagram (Figure 1).

### Overview of the included evidence

The final analytic dataset contained 111 studies on implementation-relevant work in Pakistan’s health sector. Most were Pakistan-only studies 93 [83.8%], while 18 (16.2%) were multi-country studies that included Pakistan.

**Figure 2.**
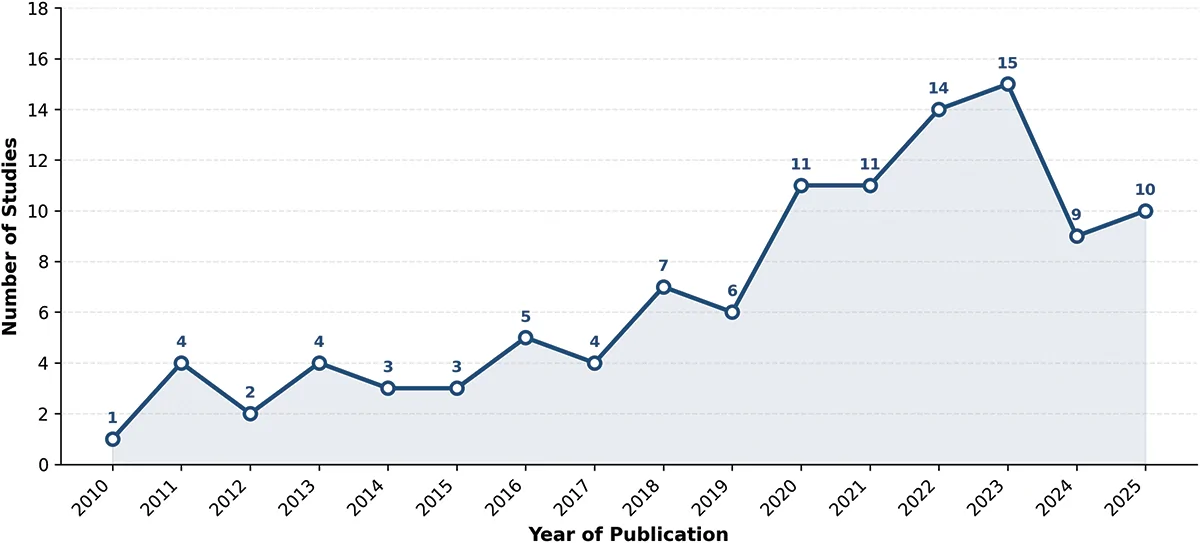
Annual trend in included sources (*n* = 111).

As shown in Figure 2, publication activity increased steadily over time, with a clear concentration from 2020 onward and a peak in 2023 (*n* = 15).

### Study characteristics and methodological profile

Methodologically, the literature was heterogeneous but leaned toward applied and practice-facing designs. Mixed-methods studies were most common (36.0%), followed by qualitative studies (18.9%) and other designs including quality improvement projects, clinical audits, and programme evaluations (14.4%). Quantitative studies accounted for 11.7%, followed by policy and programme analyses (6.3%), randomised controlled trials (6.3%), case studies (3.6%), and reviews (2.7%). Table 2 presents the full study design distribution.

**Table 2.** Study design distribution.

Study design	n	%
Mixed methods	40	36.0
Qualitative	21	18.9
Other	16	14.4
Quantitative	13	11.7
Policy/programme analysis	7	6.3
RCT	7	6.3
Case study	4	3.6
Review	3	2.7

Note: Percentages may not sum to exactly 100% because of rounding. The “Other” category (*n* = 16) comprised quality improvement projects (*n* = 3), pre-post interventional studies (*n* = 3), programme evaluations (*n* = 3), quasi-experimental designs (*n* = 2), clinical audits (*n* = 1), and other applied research designs (*n* = 4).

### Topical focus and implementation settings

As shown in Table 3, the evidence base was concentrated in maternal, newborn and child health (30.6%) and infectious diseases (24.3%), with smaller but visible clusters in other health domains (11.7%), mental health (10.8%), universal health coverage and broader health systems work (9.9%), and non-communicable diseases (9.9%). Digital health appeared as a primary domain in only three studies (2.7%).

**Table 3.** Major health domains.

Health domain	n	%
MNCH	34	30.6
Infectious diseases	27	24.3
Other	13	11.7
Mental health	12	10.8
UHC/health systems	11	9.9
NCDs	11	9.9
Digital health	3	2.7

Note: Percentages are based on the full analytic dataset (*N* = 111). The 13 studies in the “Other” domain category spanned health systems governance (*n* = 4), health professions education (*n* = 3), adolescent reproductive health, paediatric rehabilitation, nutrition, immunisation logistics, family planning, and HIV surveillance.

### Use of implementation science frameworks

Overall, 107 of 111 records (96.4%) referenced an organizing framework, logic model, or conceptual approach of some kind (Table 4). Among named implementation science frameworks, CFIR was most common (13.5%), followed by RE-AIM (7.2%), and isolated use of PRISM, PARIHS, NPT, and AIF. For example, one CFIR-informed study examined how service-delivery implementation strategies drawn from the polio eradication programme could be adapted to advance progress towards universal health coverage [16]. A RE-AIM-informed evaluation similarly assessed the reach, effectiveness, adoption, implementation, and maintenance of essential health service packages across multiple low- and middle-income health systems, including Pakistan [17]. The largest category, however, was ‘other/local or adapted’ frameworks (77.5%). This category was heterogeneous and, as shown in Supplementary Table S2, included programme-specific implementation approaches, locally developed conceptual models, adapted logic models or programme theories, operational delivery and supervision frameworks, systems or governance models, behavioral models, community implementation approaches, and implicit conceptual approaches that were not formally named. In many studies, these approaches were used pragmatically to guide implementation, adaptation, service delivery, or evaluation, but were not explicitly positioned within the formal implementation science literature. An embedded implementation-research approach combined routine programme data with structured problem-solving cycles at the district level to identify and address service-delivery bottlenecks within Pakistan’s Expanded Programme on Immunisation [18]. This pattern suggests that implementation-oriented work in Pakistan is often conceptually structured, but frequently under-labelled or only partially aligned with standardized implementation science taxonomies.

**Table 4.** Frameworks used in the included literature.

Category	n	%
Other/local or adapted framework	86	77.5
CFIR	15	13.5
RE-AIM	8	7.2
No explicit framework reported	4	3.6
PRISM	2	1.8
PARIHS	1	0.9
AIF	1	0.9
NPT	1	0.9

Note: Counts are based on binary coding; records may contribute to more than one framework category.

### Implementation strategies and outcomes

**Figure 3.**
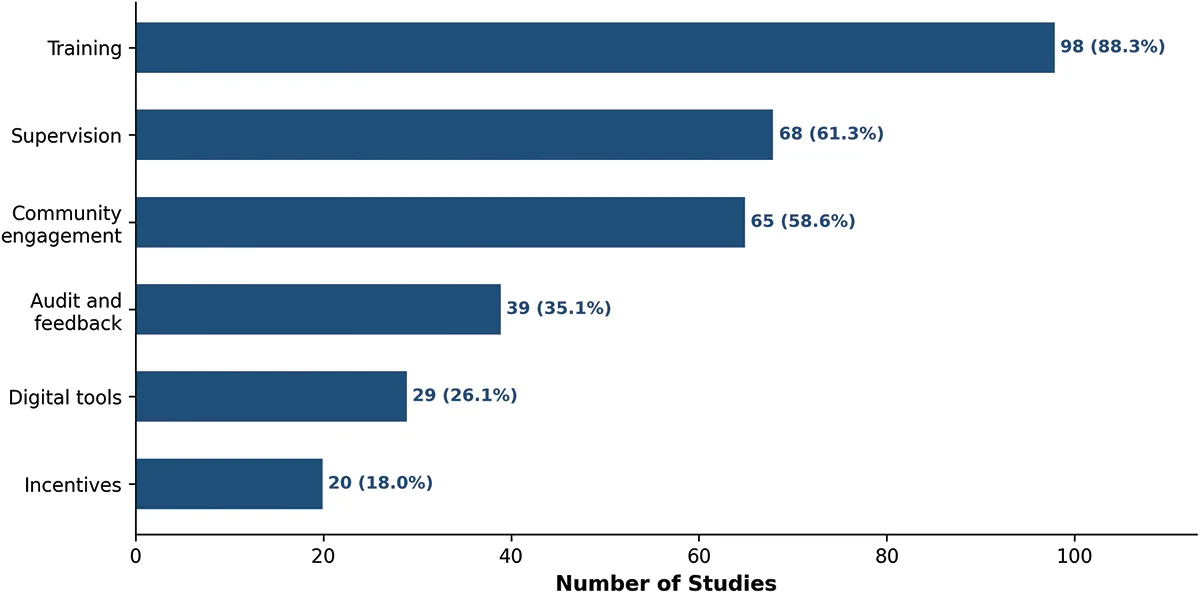
Most frequently reported implementation strategies (*n* = 111).

Training was the dominant strategy (88.3%), followed by supervision (61.3%), community engagement (58.6%), audit and feedback (35.1%), digital tools (26.1%), and incentives (18.0%) as shown in Figure 3. A multi-country tobacco-cessation programme embedded within routine tuberculosis services that relied heavily on health-worker training [19], and a supportive supervision intervention for lady health supervisors designed to strengthen frontline worker performance in rural Pakistan [20]. Community engagement was central to a voucher-based programme that reduced financial and informational barriers to long-acting reversible contraception in underserved communities in Yemen and Pakistan [21]; audit and feedback underpinned a hospital-based balanced-scorecard initiative used to track and improve performance across multiple service domains [22]; and digital tools were exemplified by a pragmatic cluster-randomised trial delivering non-communicable disease management through mobile-enabled community health workers during the COVID-19 pandemic in rural Pakistan [23].

**Figure 4.**
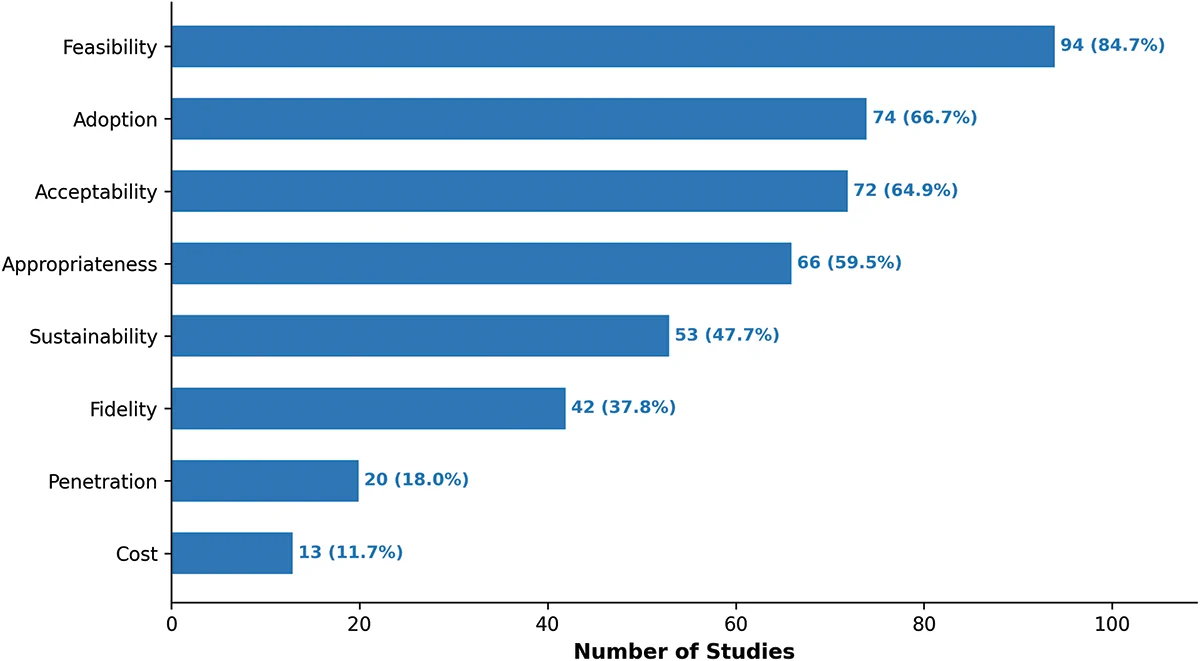
Implementation outcomes reported across included sources (*n* = 111).

Feasibility was most frequently reported (84.7%), followed by adoption (66.7%), acceptability (64.9%), appropriateness (59.5%), and sustainability (47.7%). For instance, feasibility was central to the qualitative work guiding adaptation of the FAST-M maternal sepsis intervention for Pakistani facility settings [24]. Adoption was illustrated by hospital-wide uptake of core antibiotic stewardship elements across tertiary care facilities in Punjab [25], while acceptability was evident in caregiver-reported experiences of a parent-delivered early intervention programme for children with Down syndrome [26]. Fidelity was reported in over one-third of studies (37.8%), while penetration (18.0%) and cost (11.7%) were less common (Figure 4).

**Figure 5.**
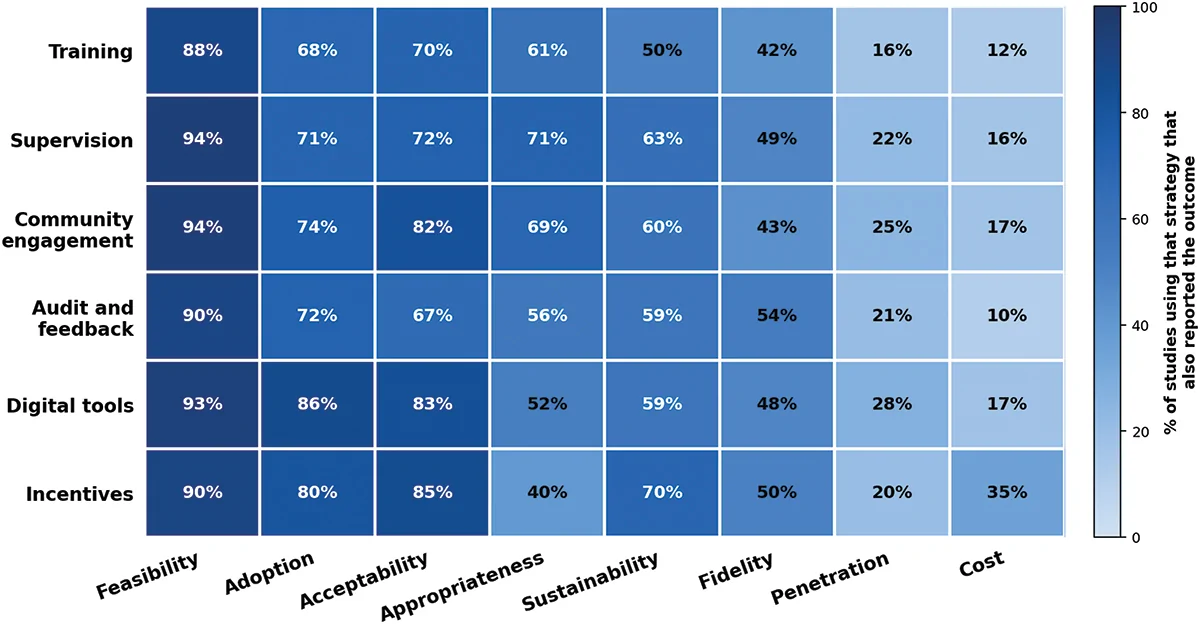
Cross tabulation of implementation strategies and outcomes (*n* = 111).

According to Figure 5, across all six strategies, feasibility was the most frequently co-reported outcome, ranging from 87.8% of training studies to 94.1% of supervision studies, followed by acceptability and adoption (66.7–86.2%). Cost and penetration were the least frequently co-reported outcomes for most strategies, with cost co-reporting below 20% for training (12.2%), audit and feedback (10.3%), supervision (16.2%), community engagement (16.9%), and digital tools (17.2%). Incentive-based strategies were the exception, with the highest cost co-reporting of any strategy (35.0%) but the lowest appropriateness co-reporting (40.0%). Supervision and community engagement showed the most balanced profiles across outcomes, while training, despite being the most frequently reported strategy overall (88.3% of studies), showed comparatively lower co-reporting of acceptability, appropriateness, and fidelity relative to these two strategies.

### Determinants of implementation: barriers, challenges, and facilitators

As given in Table 5, system-level barriers dominated (95.5%), followed by financial (63.1%), cultural (60.4%), and policy barriers (53.2%). System-level constraints of this kind were exemplified by a mixed-methods study of routine immunisation delivery in the peri-urban slums of Karachi, which documented cold-chain, staffing, and micro-planning bottlenecks at the facility level [27]. Financial constraints were similarly evident in efforts to scale up community-based management of possible serious bacterial infections in young infants when referral was not feasible [28], while cultural and geographic barriers were documented in an analysis of how social norms and mobility restrictions shaped lady health workers’ reach and effectiveness [29].

**Table 5.** Main implementation barrier categories.

Category	n	%
System barriers	106	95.5
Financial barriers	70	63.1
Cultural barriers	67	60.4
Policy barriers	59	53.2
Capacity barriers	16	14.4
Environment/security barriers	1	0.9

Note: Percentages are based on the full analytic dataset (*N* = 111). Barrier categories were coded as non-mutually exclusive.

The most frequent facilitators were training (86.5%), leadership (76.6%), funding (45.0%), technology (38.7%), and community engagement (18.0%), as given in Table 6. This was shown by a leadership-development intervention that improved family experience of care in a private pediatric hospital [30]

**Table 6.** Main implementation facilitator categories.

Category	n	%
Training as facilitator	96	86.5
Leadership	85	76.6
Funding	50	45.0
Technology	43	38.7
Community engagement	20	18.0
Stakeholder collaboration	6	5.4
Supervision	3	2.7
Governance/policy support	1	0.9

Note: Percentages are based on the full analytic dataset (*N* = 111). Facilitator categories were coded as non-mutually exclusive.

### Active implementation framework interpretation of the Pakistan landscape

When interpreted through the Active Implementation Framework, the Pakistan literature reveals a field that is rich in implementation practice but uneven in implementation theory (Table 7). The review shows strong attention to usable innovations: intervention packages were typically operationalized through concrete components such as training, supervision, community engagement, audit systems, or digital supports. The literature spans multiple implementation stages, especially installation and early implementation at facility, district, and national levels, but fewer studies robustly trace later-stage scale-up, institutionalization, or sustainment. Implementation drivers are highly visible. Competency drivers are reflected in the centrality of training and supervision; organizational drivers appear in leadership, technology, and funding; and systems drivers appear in the recurring role of government, policy, and donor environments. Improvement cycles are present but weakly named. Audit and feedback were common, yet explicit PDSA framing was rare, suggesting a gap between practice-based learning and formal improvement-science articulation. Finally, system intervention is a defining feature of the landscape: implementation in Pakistan is not carried out by a single actor, but by interacting government, community, NGO, donor, academic, and sometimes private-sector arrangements. In that sense, the literature portrays Pakistan not as a context where implementation science is absent, but as one where implementation science often exists in applied, hybrid, and under-labelled forms.

**Table 7.** Interpreting the review through the Active implementation framework.

AIF domain	What the Pakistan evidence suggests	Signals from the review
Usable innovations	Most studies described intervention packages in concrete operational terms, usually combining training, supervision, community engagement, and locally adapted delivery tools.	Training 98 (88.3%); supervision 68 (61.3%); community engagement 65 (58.6%); feasibility 94 (84.7%).
Implementation stages	Evidence spanned multiple levels of delivery, suggesting attention to installation and initial implementation at facility, district, and national levels, with fewer studies tracing later-stage scale-up or sustainment.	Sustainability outcome 53 (47.7%).
Implementation drivers	Competency and organisational drivers were highly visible: training, leadership, funding, and technology repeatedly emerged as enablers, while system, financial, and policy bottlenecks constrained delivery.	Facilitators: training 96 (86.5%), leadership 85 (76.6%), funding 50 (45.0%); barriers: system 106 (95.5%), financial 70 (63.1%), policy 59 (53.2%).
Improvement cycles	Iterative learning was present but under-labelled. Many studies used audit, feedback, supervision, or embedded learning, yet very few explicitly framed this work as PDSA or formal improvement cycles.	Audit and feedback 39 (35.1%); explicit PDSA/QI 1 (0.9%).
System intervention	Implementation in Pakistan was embedded in a pluralistic governance ecosystem involving government, academia, communities, NGOs, donors, and, less commonly, the private sector.	Government 88 (79.3%); academia 71 (64.0%); community 66 (59.5%); NGOs 49 (44.1%); donors 47 (42.3%).

Note: This table is an interpretive synthesis. Only one included study explicitly named AIF; the mapping therefore reflects review-level interpretation rather than direct author self-classification.

## Discussion

This scoping review provides a systematic mapping of implementation science and implementation-related research conducted within Pakistan’s health sector. The findings indicate that while implementation-oriented research is increasingly present in the literature, the field remains uneven in its application of formal implementation science frameworks and systematic evaluation approaches. Across the 111 studies included in this review, implementation work was largely focused on understanding program delivery, contextual bottlenecks, and the feasibility of interventions within real-world health systems. These patterns suggest that implementation research in Pakistan has evolved primarily as a pragmatic response to operational challenges faced by health programs rather than as a formally structured field grounded in implementation science theory.

A key observation from the review is that the literature is strongly oriented toward understanding how health interventions function in practice. Many studies examined issues such as service delivery constraints, provider and community acceptance of interventions, and the contextual factors influencing implementation outcomes. This orientation reflects the broader objectives of implementation research, which seeks to understand how evidence-based interventions can be integrated into routine health systems [2,31]. Similar patterns have been reported in other low- and middle-income countries, where implementation research often develops alongside efforts to scale health interventions within resource-constrained systems [32]. As discussed further below, several of the specific patterns identified in this review – including a heavy reliance on training and supervision as an implementation strategy, and limited explicit use of frameworks such as CFIR and RE-AIM – recur across other LMIC settings, though not uniformly, suggesting that some features reflect broader health-system structural conditions while others remain more context-specific to Pakistan.

The distribution of studies across health domains further illustrates how implementation research in Pakistan has been shaped by national and global health priorities. A large proportion of the literature focused on maternal, newborn, and child health (MNCH) and infectious disease programs. Within MNCH, studies addressed implementation challenges related to maternal sepsis management and adaptation of service delivery interventions within real-world care settings [24]. Infectious disease and pandemic-response implementation research in the included literature examined integration of tobacco cessation within tuberculosis programmes as well as the role of policy actors in implementing social distancing during the COVID-19 response in Pakistan [19,33]. These findings are consistent with global implementation research trends in which disease control programs often serve as entry points for applied implementation research [31].

Mental health implementation research was also represented within the literature, although the field remains comparatively smaller. Studies in this area explored the acceptability, appropriateness, feasibility, and implementation design of technology-assisted mental health interventions delivered through community frontline workers, particularly lady health workers, in rural Pakistan [34,35]. This emerging body of work reflects an increasing recognition of the importance of integrating mental health services into primary care and community health systems, particularly in low-resource settings [36].

Despite the growing volume of implementation-related studies, explicit use of implementation science frameworks was limited. Many studies discussed implementation processes, barriers, and facilitators without explicitly situating their analyses within established frameworks such as the Consolidated Framework for Implementation Research (CFIR) or RE-AIM. Similar limited engagement with named frameworks has been reported in South Asia, where India’s ASHA community health worker programme has rarely been evaluated using CFIR or RE-AIM despite structurally comparable service-delivery arrangements [37], and in Sub-Saharan Africa, where recent critical analyses argue that limited explicit framework use reflects a deeper mismatch between these frameworks’ assumptions and the relational, community-governance-based mechanisms that actually drive successful implementation, rather than a lack of conceptual rigor [38]. In Latin America, by contrast, CFIR and RE-AIM have required deliberate translation and adaptation for regional use, a pattern attributed to these frameworks’ global-north origins [39]. In some cases, these studies used conceptual models that were conceptually related to implementation science but did not explicitly identify themselves as implementation science research. For example, studies referencing the Active Implementation Framework have highlighted the importance of implementation stages and system drivers in program delivery [4]. This pattern is not unique to Pakistan: a systematic review of CFIR applications across low- and middle-income countries found that framework constructs are frequently adapted, partially applied, or invoked post hoc to interpret findings rather than prospectively used to guide study design, indicating that uneven or informal framework uptake is a broader feature of implementation science practice in LMIC settings [40]. This is corroborated by a recent scoping review of implementation science frameworks applied to primary health care interventions across LMICs, which found that RE-AIM was explicitly applied in only seven of eighteen included studies, with the remainder relying on other or unnamed frameworks – a pattern of partial and inconsistent framework uptake closely mirroring that observed in the present review [41]. However, broader implementation science literature suggests that systematic use of theoretical frameworks can strengthen analytic rigor and improve comparability across studies [3,42].

This tension shows up in our own data. Implementation in Pakistan rarely rested with a single, institutionally bounded actor of the kind CFIR, RE-AIM, and AIF implicitly assume government, academic, community, NGO, and donor actors were involved in overlapping proportions across studies (79.3%, 64.0%, 59.5%, 44.1%, and 42.3%, respectively; Table 7), with implementation responsibility and financing distributed across parallel, donor-dependent project cycles rather than one accountable system. Against this backdrop, the limited measurement of cost (11.7%) and sustainability (47.7%) outcomes may reflect not only a methodological gap but a conceptual mismatch: ‘maintenance,’ ‘sustainment,’ and cost-effectiveness all presuppose a single organizational home and a single budget holder, assumptions poorly suited to fragmented, externally financed delivery. This links our findings to the broader debate on decolonizing and diversifying implementation science, in which Western-designed frameworks are argued to encode institutional and funding assumptions that can misread genuine contextual adaptation as a lack of rigor [38,43]. Seen this way, the 77.5% of studies using ‘other/local or adapted’ frameworks may reflect not a conceptual shortcoming but locally adapted operational models made necessary by a genuinely different implementation landscape – a pattern meriting further theory-building grounded in local health-system experience.

The implementation strategies reported across the studies were relatively consistent. Most interventions relied on a combination of capacity building, community engagement, and supervisory support mechanisms. These strategies reflect efforts to strengthen human capability within the health workforce, promote community acceptance of health programs, and maintain delivery quality through monitoring and supervision structures. This reliance on training and supervision mirrors patterns reported elsewhere in LMICs: South Asia’s ASHA community health worker programme has similarly depended heavily on training and supervisory strategies [37], and a multi-country synthesis spanning Africa, Asia, and Latin America identified training, supervision, and workload as structural determinants of community health worker performance across resource-constrained health systems generally, rather than a Pakistan-specific pattern [44]; a broader review of implementation-research innovations across these same regions similarly emphasises context-embedded, participatory approaches over rigid application of standardized strategies [45]. Such approaches correspond closely with commonly described implementation strategies in the broader literature [31,46]. At the same time, formal quality improvement approaches such as Plan–Do–Study–Act cycles or structured improvement collaboratives were rarely reported, suggesting that quality improvement terminology and methods remain underutilized within implementation research conducted in Pakistan. The prominence of ‘other/local or adapted’ frameworks indicates that implementation work in Pakistan is often conceptually structured, but not consistently articulated using formal implementation science terminology.

The pattern of implementation outcomes reported in the literature further reinforces this interpretation. Feasibility, acceptability, and adoption were the most frequently assessed outcomes, indicating strong attention to early and mid-stage implementation processes. In contrast, outcomes related to penetration, long-term sustainability, and economic evaluation were reported much less frequently. This distribution aligns with broader observations in implementation science, where early implementation outcomes are more commonly measured than long-term system outcomes such as sustainment or cost-effectiveness [13,42]. Strengthening the measurement of these later-stage outcomes will be important for understanding how health interventions scale and become institutionalized within routine health systems.

Viewed jointly, the pattern suggests that evaluation depth tracks strategy type more than strategy frequency: supervision and community engagement, though used less often than training, were evaluated against a broader, more balanced set of outcomes. This is consistent with multi-country evidence positioning supervision and community-level engagement, rather than training alone, as the more consistently emphasised determinants of implementation performance across African, Asian, and Latin American health systems [44]. Training’s dominance in frequency without matching breadth of evaluation may reflect its status as the default, lowest-cost strategy reached for regardless of context. Cost and penetration were rarely reported for any strategy, except for incentive-based strategies. This fits the argument that in a fragmented, donor-dependent implementation landscape, tracking cost-effectiveness or scale requires a single accountable budget holder, which rarely exists in practice [38,43].

Barriers and facilitators reported across the studies were also highly patterned. Implementation challenges were frequently linked to health system capacity constraints, workforce shortages, governance limitations, and financing gaps. These findings suggest that implementation failures are rarely attributable to a single missing factor but instead reflect interactions among organizational capacity, policy environments, and sociocultural contexts. Similar multi-level determinants of implementation success have been widely documented in implementation science literature [3,31]. The Latin American experience offers a partial contrast: analyses of implementation-research uptake in that region point to institutional and political-economy determinants – such as governance structures and the availability of local implementation-research capacity – as more decisive than frontline strategy selection, suggesting that the specific configuration of barriers and facilitators identified in this review may not generalize uniformly across all LMIC regions [47].

This review has several strengths. It provides one of the most comprehensive mappings of implementation science and implementation-related research within Pakistan’s health sector. The use of multiple databases and institutional repositories enabled the identification of a wide range of empirical studies and program evaluations. In addition, the structured data extraction framework allowed systematic mapping of implementation frameworks, strategies, outcomes, and contextual determinants. However, several limitations should be noted. This review has some limitations that should be considered when interpreting the findings. The search did not include Web of Science, Scopus, or Embase, as institutional access to these platforms was not available to the review team. Although PubMed, Google Scholar, PakMediNet, WHO IRIS/EMRO, and several Pakistani institutional repositories were searched, studies indexed exclusively in the excluded databases may not have been captured, which could underestimate the full scope of implementation-relevant research in Pakistan. Google Scholar was screened page by page until results ceased to be relevant, which occurred at approximately 200 pages per search string, while this saturation-based approach is more thorough than a fixed cutoff, it cannot be fully standardised, and some studies may still have been missed. The restriction to English-language publications is a less significant constraint in the Pakistani research context than it might be elsewhere, given that the large majority of academic and institutional health research in Pakistan is published in English and PakMediNet indexes local English-language journals; nonetheless, some grey literature and government reports produced primarily for domestic use may not have been captured. The review protocol was not prospectively registered, which reduces transparency and cannot exclude the possibility of post hoc modifications to eligibility or analytical decisions, though the approach reported here is consistent with the protocol developed prior to the search. Classification of studies as implementation-relevant required interpretive judgement, introducing some subjectivity that was mitigated through independent dual screening and third-reviewer verification of the random sample. Finally, consistent with scoping review methodology, no formal quality appraisal of individual studies was undertaken; the findings therefore map the breadth of the implementation science landscape in Pakistan rather than the strength of the underlying evidence base. A further limitation concerns the interpretive framework used to synthesise findings: the Active Implementation Framework (AIF) represents a structure imposed by the review team rather than one emerging from the primary studies themselves, and a synthesis organised instead around CFIR’s determinant domains might have foregrounded different findings, such as contextual barriers, rather than process stages and system-level capacity.

The findings of this review have several implications for research and policy. First, greater use of established implementation science frameworks could strengthen the rigor and comparability of implementation research conducted in Pakistan. Second, implementation science approaches should be expanded beyond traditional program areas such as MNCH and infectious diseases to address emerging health challenges including noncommunicable diseases, mental health services, and digital health systems. Third, future research should place greater emphasis on long-term outcomes such as sustainability, institutionalization, and economic evaluation of health interventions. Finally, integrating implementation research more explicitly into routine program design and evaluation processes may help strengthen evidence-informed decision-making and improve the translation of health policies into effective service delivery.

## Conclusions

This review highlights a growing but still evolving body of implementation-oriented research within Pakistan’s health sector. While studies frequently address feasibility and contextual challenges, greater use of implementation science frameworks, stronger evaluation of sustainability and scale, and expanded research across emerging health priorities are needed to strengthen evidence-informed health system implementation.

## Supplementary Material

Supplemental Material

## Data Availability

Supplementary materials associated with this scoping review, including the search strategies, data extraction tables, framework classifications, and PRISMA-ScR checklist, are publicly available on Zenodo at: https://doi.org/10.5281/zenodo.22184997.
