# Supplementary material for: Mapping implementation science in Pakistan’s health sector: a scoping review of frameworks, strategies, and implementation outcomes
Source: Glob Health Action. 2026 Sep 16;19(1):2732675. doi: 10.1080/16549716.2026.2732675 (PMC13587568; doi:10.1080/16549716.2026.2732675)
Supplement: Supplementary file 1 [file ZGHA_A_2732675_SM6183.zip › Supplementary Files/0830_Supplementary_File_S3_N111.docx]

# **Supplementary File S3. Search Strategy**

**Manuscript:** *Mapping Implementation Science in Pakistan's Health Sector: A Scoping Review of Frameworks, Strategies, and Implementation Outcomes*

**Journal:** Global Health Action | Ms. No. 262218598

**Corresponding author:** Dr Sheh Mureed, Ministry of Planning, Development and Special Initiatives, Islamabad

An initial search was conducted in July 2025. Following peer review, the PubMed search strategy was expanded in June 2026 as recommended by Reviewer 1, incorporating additional implementation science terminology, full framework names alongside acronyms, MeSH terms, and Pakistan* truncation. Both search strings are reported below. The combined deduplicated PubMed pool from both searches is 334 unique records. Across all sources, 460 records were screened against the eligibility criteria. Following re-application of the restructured PCC eligibility criteria, 111 studies were included in the final review (see PRISMA-ScR flow, Figure 1 of the main manuscript).

## **1. PubMed**

**a) Original search — 17 July 2025 | Records retrieved: 144**

**Block 1 — Implementation science concepts [Title/Abstract]**

"implementation science" OR "implementation research" OR "implementation framework*" OR "implementation strateg*" OR "implementation outcome*" OR "process evaluation" OR "program implementation" OR "programme implementation" OR "knowledge translation" OR adoption OR feasibility OR fidelity OR acceptability OR penetration OR sustainability OR "scale-up" OR "scale up" OR barrier* OR facilitator* OR "quality improvement" OR PDSA OR "active implementation" OR CFIR OR "RE-AIM" OR EPIS OR PARIHS OR PRISM OR NPT

**Block 2 — Health sector concepts [Title/Abstract]**

health OR "health sector" OR "health system*" OR "healthcare delivery" OR "health program*" OR "health programme*" OR "health intervention*" OR hospital* OR clinic* OR "primary care" OR "public health" OR MNCH OR nutrition OR immunization OR immunisation OR tuberculosis OR HIV OR "mental health" OR NCD*

**Block 3 — Geography [Title/Abstract and MeSH]**

Pakistan[Title/Abstract] OR Pakistan[MeSH Terms]

*Filters: English language; Publication date 01/01/2010 to 30/06/2026*

**b) Expanded search — June 2026 | Records retrieved: 280 | Combined unique pool: 334**

The following additions were made in response to Reviewer 1 comments:

Additional IS terms: "knowledge translation", "research utilization", "research utilisation", "evidence uptake", "evidence-based practice", "dissemination research", "implementation interventions"

Full framework names alongside acronyms: "Consolidated Framework for Implementation Research", "Exploration Preparation Implementation Sustainment", "Promoting Action on Research Implementation in Health Services", "Practical Robust Implementation and Sustainability Model", "Normalization Process Theory"

MeSH terms: "Implementation Science"[MeSH], "Diffusion of Innovation"[MeSH], "Quality Improvement"[MeSH], "Program Evaluation"[MeSH]

Pakistan truncation: Pakistan*[Title/Abstract]

**Expanded PubMed string (June 2026):**

("implementation science"[Title/Abstract] OR "implementation research"[Title/Abstract] OR "implementation framework"[Title/Abstract] OR "implementation strategy"[Title/Abstract] OR "implementation strategies"[Title/Abstract] OR "implementation outcome"[Title/Abstract] OR "implementation outcomes"[Title/Abstract] OR "implementation intervention"[Title/Abstract] OR "process evaluation"[Title/Abstract] OR "program implementation"[Title/Abstract] OR "programme implementation"[Title/Abstract] OR "knowledge translation"[Title/Abstract] OR "research utilization"[Title/Abstract] OR "research utilisation"[Title/Abstract] OR "evidence uptake"[Title/Abstract] OR "evidence-based practice"[Title/Abstract] OR "dissemination research"[Title/Abstract] OR "implementation fidelity"[Title/Abstract] OR "implementation sustainability"[Title/Abstract] OR "implementation feasibility"[Title/Abstract] OR "scale-up"[Title/Abstract] OR "scale up"[Title/Abstract] OR "quality improvement"[Title/Abstract] OR "active implementation"[Title/Abstract] OR CFIR[Title/Abstract] OR "Consolidated Framework for Implementation Research"[Title/Abstract] OR EPIS[Title/Abstract] OR "Exploration Preparation Implementation Sustainment"[Title/Abstract] OR PARIHS[Title/Abstract] OR "Promoting Action on Research Implementation in Health Services"[Title/Abstract] OR PRISM[Title/Abstract] OR "Practical Robust Implementation and Sustainability Model"[Title/Abstract] OR NPT[Title/Abstract] OR "Normalization Process Theory"[Title/Abstract] OR "RE-AIM"[Title/Abstract] OR "Implementation Science"[MeSH] OR "Diffusion of Innovation"[MeSH] OR "Quality Improvement"[MeSH] OR "Program Evaluation"[MeSH]) AND ("health system"[Title/Abstract] OR "health sector"[Title/Abstract] OR "healthcare"[Title/Abstract] OR "health program"[Title/Abstract] OR "health programme"[Title/Abstract] OR "health intervention"[Title/Abstract] OR "primary care"[Title/Abstract] OR "public health"[Title/Abstract] OR "community health"[Title/Abstract] OR MNCH[Title/Abstract] OR tuberculosis[Title/Abstract] OR HIV[Title/Abstract] OR "mental health"[Title/Abstract] OR immunization[Title/Abstract] OR immunisation[Title/Abstract]) AND (Pakistan[Title/Abstract] OR Pakistan*[Title/Abstract] OR Pakistan[MeSH Terms]) AND ("2010/01/01"[Date - Publication] : "2026/06/30"[Date - Publication]) AND english[Language]

## **2. Google Scholar**

**Date searched:** 17-18 July 2025 | Records carried forward: 74 (from ~6,160 raw hits)

Google Scholar was searched using multiple targeted strings combining implementation science concepts with Pakistan and specific health domain terms. Results were screened page by page until saturation (no further relevant results), which occurred at approximately 200 pages per string. Relevant records were carried forward (n = 74).

**Representative search strings:**

"implementation science" Pakistan health "implementation research" Pakistan "implementation framework" Pakistan health sector "quality improvement" Pakistan health "process evaluation" Pakistan "scale-up" health intervention Pakistan "knowledge translation" Pakistan health CFIR Pakistan | RE-AIM Pakistan health barriers facilitators implementation Pakistan

## **3. PakMediNet**

**Date searched:** 18 July 2025 | Records retrieved: 26 | Screened: 18 | Included: 4

Search strings (run separately):

Implementation science Pakistan → 12 hits; 8 screened; 2 included Program evaluation health Pakistan → 8 hits; 6 screened; 1 included Knowledge translation Pakistan → 6 hits; 4 screened; 1 included

## **4. WHO IRIS / WHO EMRO**

**Refined search:** 19 July 2025 | Records retrieved: 45 | Screened: 20 | Included: 5

A refined combined string was used to identify implementation-relevant documents specific to Pakistan's health sector. A broader export (~500 records, 22 July 2025) was also retrieved; the 20 most relevant records were taken forward for screening.

**Core search terms:**

("implementation science" OR "implementation research" OR "program evaluation" OR "process evaluation") AND "health" AND "Pakistan" implementation AND Pakistan AND health "implementation research" AND Pakistan "health system" AND implementation AND Pakistan

## **5. Pakistani Institutional Repositories**

**Date searched:** July 2025 | Records carried forward: 14 (entries 75-88 of the combined screening list)

Repositories: Aga Khan University (AKU); Health Services Academy (HSA), Islamabad; University of Health Sciences (UHS), Lahore.

**Search terms:** implementation, implementation research, health system, programme evaluation, Pakistan

## **6. Manual and Citation Searching**

Additional records were identified through: (a) reference list scanning of key included studies; (b) forward citation searching of seminal implementation science papers; and (c) review of grey literature including WHO Pakistan country office reports and Ministry of National Health Services publications.

## **Summary of Search Results and Screening by Source**

*All figures are drawn directly from annotated search logs, colour-coded screening files, or summary logs maintained during the review.*

| **Source** | **Search date** | **Records identified** | **Records screened** | **Records included** |
| --- | --- | --- | --- | --- |
| PubMed (original) | 17 Jul 2025 | 144 | 144 | 62 |
| PubMed (expanded) | Jun 2026 | 280 (190 new) | 190 | 11 |
| WHO IRIS / EMRO | 19 Jul 2025 | 45 | 20 | 5 |
| PakMediNet | 18 Jul 2025 | 26 | 18 | 4 |
| Google Scholar | Jul 2025 | ~6,160 | 74 | - |
| Institutional & manual | Jul 2025 | - | 14 | - |
| **Total** |  |  | **460** | **111** |

*Notes.*

• "Records screened" = records assessed against eligibility criteria. For Google Scholar this is records carried forward after saturation screening, not the total hits returned.

• Per-source included counts verified from colour-coded screening file (PubMed: 62+11=73) and annotated summary logs (WHO IRIS: 5, PakMediNet: 4). The remaining included studies were drawn from the Google Scholar and institutional/manual pool.

• Following Reviewer 1's recommendation, the eligibility criteria were restructured using the PCC framework and re-applied to all records. Of the 460 records screened by title and abstract, 258 were excluded for not meeting the eligibility criteria, leaving 202 full-text records assessed for eligibility; a further 91 were excluded at full-text review due to lack of relevance to Pakistan's health sector, absence of implementation-related content, non-research publication type, or inaccessibility of full text, resulting in a final dataset of N = 111 (see PRISMA-ScR flow diagram, Figure 1 of the main manuscript).

• Title and abstract screening performed independently by two reviewers (SM, SH); conflicts resolved by discussion with a third reviewer (SMur). Decisions recorded in a shared colour-coded screening log.

• Reference manager: Mendeley. Deduplication: by PMID (PubMed); manual title/author check (other sources).

• See PRISMA-ScR flow diagram (Figure 1, main manuscript) for the full study selection process.
