# Supplementary material for: Mapping implementation science in Pakistan’s health sector: a scoping review of frameworks, strategies, and implementation outcomes
Source: Glob Health Action. 2026 Sep 16;19(1):2732675. doi: 10.1080/16549716.2026.2732675 (PMC13587568; doi:10.1080/16549716.2026.2732675)
Supplement: Supplementary file 1 [file ZGHA_A_2732675_SM6183.zip › Supplementary Files/0830_Supplementary_File_S4_N111.docx]

**Supplementary File S4. PRISMA-ScR Checklist**

*Preferred Reporting Items for Systematic reviews and Meta-Analyses extension for Scoping Reviews (PRISMA-ScR) checklist for the manuscript "Mapping Implementation Science in Pakistan's Health Sector: A Scoping Review of Frameworks, Strategies, and Implementation Outcomes" (Ms. No. 262218598).*

| **Section** | **Item** | **PRISMA-ScR checklist item** | **Reported on page / section location** |
| --- | --- | --- | --- |
| **Title** | 1 | Identify the report as a scoping review. | Title |
| **Abstract** | 2 | Provide a structured summary that includes background, objectives, eligibility criteria, sources of evidence, charting methods, results, and conclusions. | Abstract |
| **Introduction** | 3 | Describe the rationale for the review in the context of what is already known. | Introduction, paragraphs 1–4 |
| **Introduction** | 4 | Provide an explicit statement of the questions and objectives being addressed. | Introduction, final paragraph |
| **Methods** | 5 | Indicate whether a review protocol exists; state if and where it can be accessed; provide registration information if available. | Methods, first paragraph ("A protocol was developed a priori for this scoping review but was not formally registered.") |
| **Methods** | 6 | Specify characteristics of the sources of evidence used as eligibility criteria and provide a rationale. | Methods, eligibility criteria paragraph; Table A (PCC-structured criteria) |
| **Methods** | 7 | Describe all information sources in the search and the date most recently searched. | Methods, information sources paragraph; Supplementary File S3 |
| **Methods** | 8 | Present the full electronic search strategy for at least 1 database, including any limits used, such that it could be repeated. | Methods, search strategy paragraph (full PubMed search string in main text); Supplementary File S3 |
| **Methods** | 9 | State the process for selecting sources of evidence. | Methods, screening/selection paragraph; Figure 1 (PRISMA-ScR flow diagram) |
| **Methods** | 10 | Describe the methods of charting data from the included sources of evidence. | Methods, data charting/extraction paragraph |
| **Methods** | 11 | List and define all variables for which data were sought and any assumptions/simplifications made. | Methods, extraction template/variables paragraph |
| **Methods** | 12 | If done, provide a rationale for conducting critical appraisal of included sources; describe methods used. | Not applicable; Methods ("formal critical appraisal of individual sources was not undertaken") |
| **Methods** | 13 | Describe the methods of handling and summarising the data that were charted. | Methods, descriptive and thematic analysis paragraph (Ritchie and Spencer framework analysis); AIF interpretation paragraph |
| **Results** | 14 | Give numbers of sources screened, assessed for eligibility, and included, with reasons for exclusions; ideally use a flow diagram. | Results, first paragraph (471 records identified; 11 duplicates removed; 460 screened; 202 full-text assessed; 91 excluded; 111 studies included); Figure 1 |
| **Results** | 15 | For each source of evidence, present characteristics for which data were charted and provide the citations. | Results sections; Tables 1–6; Supplementary Table S1 |
| **Results** | 16 | If critical appraisal was done, present the data. | Not applicable |
| **Results** | 17 | For each included source of evidence, present the relevant charted data that relate to the review questions and objectives. | Results sections; Supplementary Table S1; Supplementary Table S2 |
| **Results** | 18 | Summarise and/or present the charting results as they relate to the review questions and objectives. | Results sections; Tables 1–6; Figures 1–5 |
| **Discussion** | 19 | Summarise the main results; link to the review questions and objectives; consider relevance to key groups. | Discussion |
| **Discussion** | 20 | Discuss the limitations of the scoping review process. | Discussion, limitations paragraph |
| **Discussion** | 21 | Provide a general interpretation of the results with respect to the review questions and objectives, as well as potential implications and/or next steps. | Discussion, implications and conclusions paragraphs |
| **Funding** | 22 | Describe sources of funding for the included sources of evidence and the funding for the scoping review itself. | Funding Information |

*Adapted from: Tricco AC, Lillie E, Zarin W, et al. PRISMA Extension for Scoping Reviews (PRISMA-ScR): Checklist and Explanation. Ann Intern Med. 2018;169(7):467-473.*
